# Investigation of 6-[^18^F]-Fluoromaltose as a Novel PET Tracer for Imaging Bacterial Infection

**DOI:** 10.1371/journal.pone.0107951

**Published:** 2014-09-22

**Authors:** Gayatri Gowrishankar, Mohammad Namavari, Erwan Benjamin Jouannot, Aileen Hoehne, Robert Reeves, Jonathan Hardy, Sanjiv Sam Gambhir

**Affiliations:** 1 Department of Radiology, Stanford University School of Medicine, Stanford, California, United States of America; 2 Sanofi R&D, Sanofi, Paris, France; 3 Department of Pediatrics, Stanford University School of Medicine, Stanford, California, United States of America; 4 Department of Bioengineering, Stanford University School of Medicine, Stanford, California, United States of America; Banner Alzheimer's Institute, United States of America

## Abstract

Despite advances in the field of nuclear medicine, the imaging of bacterial infections has remained a challenge. The existing reagents suffer from poor sensitivity and specificity. In this study we investigate the potential of a novel PET (positron emission tomography) tracer that overcomes these limitations.

**Methods:**

6-[^18^F]-fluoromaltose was synthesized. Its behavior *in vitro* was evaluated in bacterial and mammalian cultures. Detailed pharmacokinetic and biodistribution profiles for the tracer were obtained from a murine model.

**Results:**

6-[^18^F]-fluoromaltose is taken up by multiple strains of pathogenic bacteria. It is not taken up by mammalian cancer cell lines. 6-[^18^F]-fluoromaltose is retained in infected muscles in a murine model of bacterial myositis. It does not accumulate in inflamed tissue.

**Conclusion:**

We have shown that 6-[^18^F]-fluoromaltose can be used to image bacterial infection *in vivo* with high specificity. We believe that this class of agents will have a significant impact on the clinical management of patients.

## Introduction

The incidence of bacterial infections in hospitals worldwide has been on the rise, particularly with the emergence of several strains of resistant pathogenic bacteria (e.g., methicillin-resistant *Staphylococcus aureus*, multidrug-resistant *Streptococcus pneumoniae*, *Clostridium difficile*, etc.). It is estimated that one out of every 25 hospitalized patients in the United States will suffer from a health care associated infection (HAI) (source: center for disease control, CDC). The CDC estimates that there were an estimated 722,000 HAI in American hospitals in 2011. 72,000 of these patients died as a direct result of these infections. Positive culture from the suspected sites (tissue and/or blood) is the current gold standard in the diagnosis of bacterial infection. This method suffers from some limitations: a) it is invasive, b) it is unable to determine spread of infection and c) the time it takes to obtain results can often result in a fatality. There have been several attempts to image bacterial infection both pre-clinically and clinically summarized in recent reviews [1]–[5]. Most of the pre-clinical work was based on optical imaging using bioluminescent strains of bacteria and fluorescent probes [6]–[9]. These studies have been valuable to medical microbiology and have helped understand several aspects of bacterial colonization, spread and susceptibility to available treatment methods. However these methods are not yet amenable to clinical translation. Clinically there have been several PET and SPECT tracers tested [1], [2], [4], [10]. The most promising of these was the study by Diaz et al using positron emission tomography (PET) and 1-2′-deoxy-2′-fluoro-β-D-arabinofuranosyl)-5-[^124^I] iodouracil (FIAU), to identify bacterial lesions in patients with suspected musculoskeletal infections [11]. The main limitation of this study is the fact that FIAU is a substrate of the bacterial enzyme-thymidine kinase but also for the mammalian mitochondrial thymidine kinase, leading to an increased background imaging signal in certain tissues. Others have shown that FIAU may not be taken up by all strains of bacteria [12] including *Pseduomonas aeruginosa* which is responsible for 51,000 of all nosocomial infections in the USA every year. Despite the multitude of imaging agents that have been developed for infection over the last decade, there is no single probe that is able to image all classes of bacteria. For eg. ^99m^Tc-Labeled antimicrobial peptide ubiquicidin (29^th^ amino acid-41^th^ amino acid) accumulates less in *Escherichia coli* infection than in *Staphlococcus aureus* infection [13]. There is therefore a need to develop a probe that would be able to image infections caused by all classes of bacteria to minimize false negatives. In a recent study, Murthy et al. have demonstrated the use of fluorescent maltodextrin-based probes to image bacteria in pre-clinical models with a high degree of specificity and sensitivity [14]. Maltose and maltodextrins appear to be used as energy sources exclusively by bacteria. All species of bacteria including pathogenic strains express a series of genes (commonly known as the maltodextrin transport complex) that accomplish the binding, transport and utilization of maltose and maltodextrin [15]–[17]. This transport mechanism is absent in mammalian cells, making maltose a substrate unique to bacteria and hence a promising choice to build imaging probes directed specifically against bacteria. PET is the most relevant imaging modality for detection of molecular signatures in the clinic because it allows whole body imaging with a sensitivity of 10^−12^ M and a high spatial resolution of ∼5 mm [18]. Here, we examine the use of a maltose based PET agent to image bacteria in cell culture and *in vivo*.

## Materials and Methods

### Synthesis

4-O-(α-D-glucopyranosyl)-6-deoxy-6-[^18^F]fluoro-D-glucopyranoside (6-[^18^F]fluoromaltose) was prepared by nucleophilic displacement of the nosylate group in **a** 1,2,3-tri-O-acetyl-4-O-(2′,3′,-di-O-acetyl-4′,6′-benzylidene-α-D-glucopyranosyl)-6-deoxy-6-nosyl-D-glucopranoside precursor by [^18^F]fluoride ion in acetonitrile at 80°C for 10 min. Initial purification of the ^18^F-labeled protected intermediate was performed via a light C-18 Sep-Pak cartridge. After passing the solution of the ^18^F-labeled intermediate in acetonitrile through a light neutral alumina Sep-Pak, it was evaporated to dryness and deprotection was carried out first with 1N HCl (110°C, 10 min) followed by 2N NaOH at room temperature for 4 min. After neutralization and HPLC purification of the solution, 6-[^18^F]fluoromaltose was recovered in 5–8% radiochemical yield (decay corrected) with 95% radiochemical purity. Additional details are available in a related manuscript [19].

### Cultures

The *E.coli* strain was obtained from American Type Culture Collections (ATCC 33456). The bioluminescent strain of *Pseudomonas aeruginosa* (Xen 5) and the *Listeria monocytogenes* strain (Xen 32) were obtained from Perkin Elmer. The mammalian cell lines MDA MB231 (breast cancer cell line), HeLa (cervical carcinoma cell line), J774 (murine macrophage cell line) and EL4 (murine lymphoma cell line), originated from ATCC and were grown in media recommended by ATCC.

### Bacterial uptake studies

An overnight (O/N) culture of the respective strain of bacteria was initiated by inoculating a colony from a plate into a 3 ml culture of LB broth. The next morning, 500 µl of the O/N culture was inoculated into 30 ml of LB in a 200 ml flask and grown in a 37°C shaker/incubator until the bacterial culture reached log phase (OD_600_ = 0.5). Aliquots of 10^8^ colony-forming units (CFU) of the bacterial culture were incubated with the tracer for the designated periods. At the end of the incubation period, unbound tracer was removed by washing and the cultures were lysed using a bacterial lysis solution (BugBuster, EMD, Billerica MA USA). The counts associated with the lysate were determined using a gamma counter. The protein concentration in the lysate was determined using standard methods (Pierce, Thermo Fisher Scientific, Rockford IL, USA). All samples were compared to total activity references and the percentage uptake per microgram protein was calculated.

### *E.coli* induced murine myositis

6–7 week old nude mice (n = 15) were anesthetized by isoflurane inhalation. 5×10^7^ CFU of a specific strain of *E.coli* (ATCC 33456) in 50 µl of LB broth was administered as an intra-muscular injection, into the right thigh of the mice. The mice were imaged 24 h after the initial infection.

### Heat inactivation of *E. coli*

An O/N culture of a bioluminescent strain of *E.coli* (Top10 harboring a plasmid encoding the *lux* operon) was set up as described above and allowed to reach log phase. Aliquots of the culture containing 10^8^ CFU of bacteria were heat inactivated at 90°C for 30 minutes and then implanted in the left thigh of nude mice (n = 3). An equal number of viable bacteria were implanted in the contralateral thigh by intra-muscular injection. 1 hour later the mice were imaged on the IVIS-200 (for bioluminescence) and micro PET/CT.

### Turpentine oil induced sterile abscess model

6–7 week old BALB/c mice (n = 3) were anesthetized by isoflurane inhalation. 30 µl of turpentine oil (Sigma) was administered intra-muscularly into the right thigh of the mice. The contralateral muscle, which received no treatment, was used as controls. The mice were imaged by micro PET/CT at 72 h post induction of inflammation. The mice were sacrificed at the end of the study for biodistribution analysis.

### Micro PET/CT

7.4MBq of the radiotracer was administered to the mice intravenously. The mice were kept anesthetized with isoflurane after tracer administration. At the desired times, the mice were placed on the bed of the micro PET/CT scanner (Inveon, Siemens, Germany) and 5 min static scans were performed. For the dynamic scans, tail vein catheters (12 cm PU tubing and 27 g butterfly needle) were inserted into the tail vein of the mice and the catheter was glued onto the tail using Vet Bond (tissue glue). Once the animal was in position in the PET part of the PET/CT scanner, 7.4MBq of the tracer was administered via the catheter and the PET scan was started. The dynamic scan was performed for an hour. During the scan special precautions were taken to make sure the mice were warm. All images were reconstructed using 3D-OSEM. Region of interest (ROI) analysis were done using IRW software (Inveon Research Workplace, Siemens, Germany).

### Biodistribution Studies

7.4MBq of the radiotracer was administered to the mice intravenously. The mice were kept anesthetized after tracer administration. At the desired time after tracer injection, the mice were sacrificed by cervical dislocation. Then relevant organs/tissues were removed, placed in gamma counter tubes and weighed. Tissue-associated radioactivity was determined in a gamma counter (Cobra, Perkin Elmer, Waltham MA USA), decay-corrected to time of tracer injection and normalized to total injected activity using diluted aliquots of the initial administered dose as standards.

### Histology

The tissues were collected in formalin, embedded in paraffin and processed. Gram staining and Hematoxlin & Eosin (H&E) staining of the muscle sections was performed using standard protocols.

### Statistics

Unpaired t test was performed to compare differences between control and treated groups using Graphpad from Prism (version 6.0, La Jolla CA, USA)

### Ethics Statement

All animal work was done as per guidelines set by the Stanford IACUC. The protocol number is #9547. The work did involve use of anesthesia (isoflurane) and the animals did have to be euthanized (cervical dislocation). Both methods were approved in the animal protocol by the Stanford IACUC.

## Results

### Cell culture studies with 6-[^18^F]-fluoromaltose

**Figure 1:**
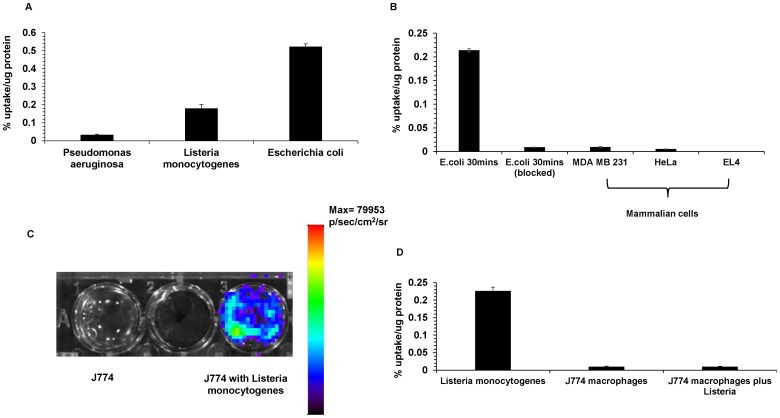
*In vitro* characterization of 6-[^18^F]-fluoromaltose. A) Uptake of 6-[^18^F]-fluoromaltose in the indicated strains of bacteria for 60 minutes. B) 1 hour uptake of 6-[^18^F]-fluoromaltose in the mammalian cell lines, MDA MB231 and HeLa and its uptake in *E.coli* in the presence of 1 mM maltose. C) Bioluminescence imaging of a macrophage cell line J774 infected with a bioluminescent strain of *Listeria monocytogenes.* D) 1 hour uptake of 6-[^18^F]-fluoromaltose in the bioluminescent strain of *Listeria monocytogenes* and in macrophage cell line J774 with and without intracellular *Listeria* infections.

The 6-[^18^F]-fluoromaltose was synthesized as described in Materials and Methods [19]. Aliquots of 10^8^ CFU of log-phase bacterial cultures (*E.coli* ATCC 33456, *P.aeruginosa* and *L.monocytogenes*) were incubated with 6-[^18^F]-fluoromaltose for the indicated times (Fig 1A). The three different strains of bacteria took up the tracer, in contrast to the tumor cell lines MDA-MB231 (human breast cancer) and HeLa (human cervical cancer) which took up very low levels of tracer (Fig 1B). As another test of the specificity of the transporter, *E.coli* cultures were incubated with and without an excess of cold maltose- the natural substrate of the maltose transporter. The uptake of 6-[^18^F]-fluoromaltose was blocked 95% (p<0.05) in the presence of 1 mM of cold maltose. In light of the results seen in Fig. 1A, we wanted to see if the 6-[^18^F]-fluoromaltose could be used to track intracellular pathogens. *L.monocytogenes* is a classic intracellular pathogen that infects macrophages. J774 a mouse macrophage cell line was infected with a bioluminescent strain of *L.monocytogenes* at a MOI of 10∶1. Fig. 1C shows that the cells have been infected with a bioluminescent strain of *Listeria*. However, the tracer is not taken up by the *Listeria* within the infected macrophages (Fig. 1D).

### Murine studies with 6-[^18^F]-fluoromaltose

**Figure 2:**
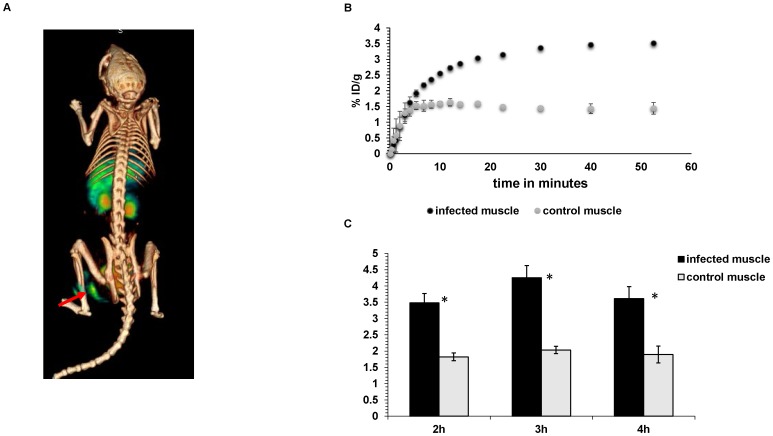
*In vivo* characterization of 6-[^18^F]-fluoromaltose. A) 3D color map from a PET/CT scan of a mouse bearing *E.coli* induced infection on the left thigh (red arrow) 1 hr after tail-vein injection of 7.4MBq of 6-[^18^F]-fluoromaltose. B) Region of interest analysis (ROIs) from PET/CT images at the indicated time points (n = 4 for each time point) * indicates statistical significance with p<0.05. C) Time activity curve showing accumulation of 6-[^18^F]-fluoromaltose in the infected muscle (n = 3).

5×10^7^ CFU of a bioluminescent strain of *E.coli* was administered into the left thigh muscle of nude mice (n = 15). 7.4MBq of the 6-[^18^F]-fluoromaltose was then administered via tail-vein. Dynamic scans were performed on some of the mice for the first 1 hr after tracer injection (Movie S1 and Fig. S3 in File S1). The remaining mice were scanned at 2 h (n = 4), 3 h (n = 4) and 4 h (n = 4) after tracer injection. Fig. 2A shows a representative 3D color map from a PET/CT scan. The tracer clearly accumulates in the infected leg. ROI analysis showed that infected muscle had an average uptake of 3.5±0.6% ID/g (mean ± SD) at 2 h, 4.2±0.7%ID/g at 3 h and 3.6±0.7% ID/g (mean ± SD) at 4 h (Fig. 2C). This is 2-fold higher than the contralateral leg at all three time points and the difference is statistically significant (p<0.05). The route of clearance of the tracer is predominantly renal with the bladder also clearly visualized, although there is hepatobiliary clearance as well. Fig. 2B and Fig. S3 in File S1 show the time-activity curves obtained from the dynamic scans. These show the tracer clearing from most organs including the control muscle. However in the infected muscle, the tracer accumulates over time and appears to be retained.

### 6-[^18^F]-fluoromaltose is taken up specifically in viable bacteria

**Figure 3:**
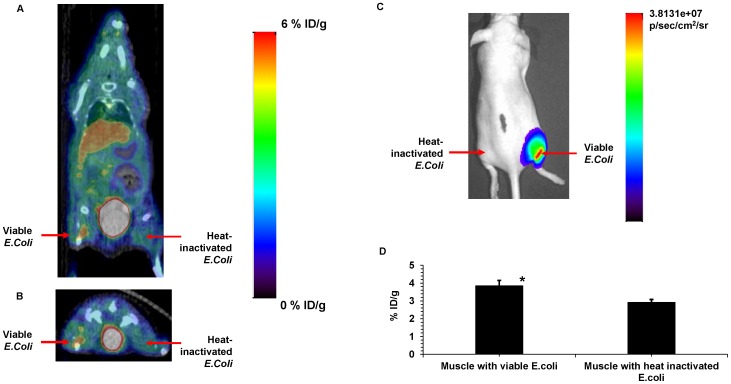
Specificity of 6-[^18^F]-fluoromaltose for viable bacteria. A) A coronal slice from a PET/CT image of a mouse bearing 10^8^ CFU of viable bioluminescent *E.coli* on the right thigh (red arrow) and 10^8^ CFU of heat-inactivated *E.coli* on the left thigh, 1hr after tail-vein injection of 7.4MBq of 6-[^18^F]-fluoromaltose B) A transverse slice from the same mouse shown in A), with arrows indicating sites of viable and heat inactivated bacteria. C) Bioluminescent image of the mouse shown in A). D) ROI analysis from PET/CT scan of mice (n = 3). * indicates statistical significance.

In order to confirm that 6-[^18^F]-fluoromaltose was taken up specifically by bacteria, 10^8^ CFU of heat inactivated bioluminescent *E.coli* was implanted in the right thigh of nude mice (n = 3). The same number of metabolically active *E.coli* was implanted in the contralateral thigh. 7.4MBq of the 6-[^18^F]-fluoromaltose was then administered 1 hour after implantation. PET/CT images show the tracer accumulating in the leg with viable bacteria (Fig. 3A). Bioluminescence imaging of the same mice (Fig. 3B) confirm the presence of viable bacteria. ROI analysis (Fig. 3C) shows that the tracer is able to differentiate tissues bearing viable bacteria from heat-inactivated bacteria (1.3 fold, p<0.05).

### 6-[^18^F]-fluoromaltose is specific for bacterial infections

**Figure 4:**
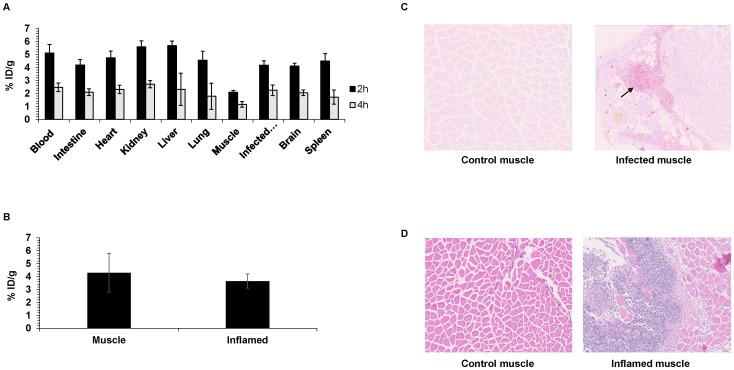
Uptake of 6-[^18^F]-fluoromaltose in infection versus inflammation. A) Ex-vivo biodistribution of 6-[^18^F]-fluoromaltose in mice bearing E.coli induced myositis, 2 h and 4 h after tail-vein injection of 7.4MBq of tracer. B) Ex-vivo biodistribution of 6-[^18^F]-fluoromaltose in mice bearing turpentine oil induced sterile abscess, 2 h after tail-vein injection of 7.4MBq of tracer. C) Representative gram stained muscle sections with a black arrow indicating presence of *E.coli* in the infected muscle section. D) Representative H&E stained muscle sections showing neutrophil infiltration in inflamed muscle.

In order to assess the specificity of the maltose tracer for bacterial infection, its uptake in the standard turpentine oil induced inflammation model was evaluated. Fig. 4A compares the ex vivo biodistribution of immunosuppressed nude mice bearing *E.coli* infection (n = 3), 24 h after infection and immunocompetent BALB/c mice bearing turpentine oil induced abscesses (n = 3) on their legs (Fig 4B), 72 h after induction. There is no difference in muscles bearing inflammation vs. the contralateral muscle in the BALB/c mice. However, in the nude mice bearing the bacterial infection, there is a 2.1 fold difference in accumulation in the infected leg vs the contralateral leg, and this difference is statistically significant (p<.05). These results indicate that the maltose class of tracers was specific for bacterial infection. Gram staining and H&E staining confirmed the presence of bacteria in infected muscles (Fig. 4C) as well as neutrophils and macrophages in the inflammation model (Fig. 4D).

## Discussion

In this paper we have evaluated 6-[^18^F]-fluoromaltose as a PET tracer for imaging bacterial infection. Our results in Fig. 1A show that this tracer is a specific substrate for the maltose family of transporters, and its uptake in *E. coli* was blocked by co-incubation with an excess of maltose. This transport system, although first identified and studied from *E.coli*, is present in multiple Gram-negative and Gram-positive bacterial species. It is not specific for pathogenic strains of bacteria and is present in all species of bacteria, although it has been shown to confer a survival advantage for certain pathogenic strains of bacteria [16], [17]. However, this class of transporters is not known to be present in mammalian cells as mentioned in the introduction. It was shown by Murthy et al. that the fluorescent maltodextrin probes did not enter mammalian cells [14].

Fig. 1B shows that 6-[^18^F]-fluoromaltose is also not taken up by multiple mammalian cell lines. ^3^H-maltose (American radiolabeled chemicals) was also taken up exclusively by bacteria and not by mammalian cell lines (Figure S1 in File S1). Hence this class of tracers (maltose and analogs) will likely image all bacterial strains with the exception of *Listeria monocytogenes* and other intracellular pathogens, which infect and colonize mammalian cells as part of their lifecycle (as shown in Fig. 1C and Fig. 1D).

In Figs. 2 and 3 the behavior of this tracer *in vivo* is shown. The tracer does accumulate specifically in infected muscle as shown in Figures 2A, B and C. The tracer also accumulates only in viable, metabolically active bacteria as shown in Fig 3A albeit with a small but significant difference to the contralateral muscle. The tracer is able to pick up as little as 10^6^ CFU of bacteria (Figure S2 in File S1), which falls within the range of the number of bacteria detected in infected tissue in a clinical setting. It does not accumulate in sites of inflammation as shown in Fig. 4B, which further demonstrates the specificity of the maltose class of probes for bacterial populations. In addition, the tracer did not accumulate in the gut microflora. However, the overall signal-to-background ratio needs to be improved in future generations of ^18^F-labeled maltose derivatives, as evident from the high blood retention (5% ID/g, Fig. 4A) and the slow elimination from other organs including liver, kidneys and brain of our first generation tracer visualized using a dynamic PET/CT scan (Figure S3 in File S1). We have evidence that this behavior might be related to the lack of complete trapping (and hence increased efflux) of 6-[^18^F]-fluoromaltose in *E.coli* cultures from cell culture studies (Figure S4 in File S1). It is not clear yet if the 6-[^18^F]-fluoromaltose is effluxed out of the bacteria as is or is partially metabolized in the bacteria into 6-[^18^F]-fluoro-6 deoxy-glucose, which cannot also be trapped [20], and is subsequently transported out of the bacteria. Both of these scenarios would explain the relatively high levels of tracer in the blood pool.

In summary we have developed and tested a novel PET tracer, 6-[^18^F]-fluoromaltose, that is taken up specifically by bacteria and only minimally taken up by mammalian cells. This specificity for bacterial cells would allow this class of probes to distinguish bacterial infection from inflammation,unlike 2′-[^18^F]- Fluoro 2′ deoxyglucose (FDG), which is taken up in both conditions. This class of tracers would image both Gram-positive and Gram-negative bacterial populations including *Pseudomonas aeruginosa*, which has been notoriously difficult to image. There are other approaches to image specific classes of bacteria but there is a need to first develop a clinically relevant approach to image all bacterial infections, akin to FDG's role in clinical oncology. Although the 6-[^18^F]-fluoromaltose does not completely fulfill these criteria because of its sub-optimal pharmacokinetics, the metabolism of maltose in man has been thoroughly investigated [21] and indicate that the pharmacokinetics of maltose based probes should be compatible with clinical development.

## Conclusions

6-[^18^F]-fluoromaltose is a promising new tracer that has been evaluated for its ability to image and localize sites of bacterial infection. Our preliminary results *in cell culture* and in mice are encouraging and we are planning additional studies to evaluate the uptake of this class of tracers in a range of clinically relevant bacterial strains and murine models. These tracers will have potential impact in the clinical management of patients suspected of having bacterial infections. Determining the spread and extent of infection particularly in the case of patients admitted with wounds or with fever of unknown origin will help guide treatment protocols and reduce the morbidity and mortality associated with sepsis.

## Supporting Information

File S1 Includes Figures S1–S4. **Figure S1**: 1 h uptake of ^3^H-maltose in bacteria (*E.coli*) and a mammalian cell line (EL 4). **Figure S2**: A) Bioluminescent image of a mouse bearing 10^6^ CFU of a bioluminescent strain of bacteria (see methods) on its right thigh B) A coronal slice from a micro PET/CT scan of the same mouse 1 h after administration of 7.4MBq of 6-[^18^F]-fluoromaltose. **Figure S3**: Time activity curve for mice (n = 3) obtained from dynamic micro PET/CT showing distribution of 7.4MBq of 6-[^18^F]-fluoromaltose in indicated organs. **Figure S4**: Plot showing 30 min uptake of 6-[^18^F]-fluoromaltose in *E.coli* and residual activity observed at 30 minutes post efflux of tracer.
(DOCX)

Movie S1 Movie showing 3D color map from a PET/CT scan of a mouse bearing an *E.coli* infection on the left leg. The 3D projection also shows tracer accumulated in the liver, kidneys and bladder.
(MPG)
