# Supplementary material for: Investigation of 6-[18F]-Fluoromaltose as a Novel PET Tracer for Imaging Bacterial Infection
Source: PLoS One. 2014 Sep 22;9(9):e107951. doi: 10.1371/journal.pone.0107951 (PMC4171493; doi:10.1371/journal.pone.0107951)
Supplement: File S1 — Includes Figures S1–S4. Figure S1: 1 h uptake of 3H-maltose in bacteria (E.coli) and a mammalian cell line (EL 4). Figure S2: A) Bioluminescent image of a mouse bearing 106 CFU of a bioluminescent strain of bacteria (see methods) on its right thigh B) A coronal slice from a micro PET/CT scan of the same mouse 1 h after administration of 7.4MBq of 6-[18F]-fluoromaltose. Figure S3: Time activity curve for mice (n = 3) obtained from dynamic micro PET/CT showing distribution of 7.4MBq of 6-[18F]-fluoromaltose in indicated organs. Figure S4: Plot showing 30 min uptake of 6-[18F]-fluoromaltose in E.coli and residual activity observed at 30 minutes post efflux of tracer. (DOCX) [file pone.0107951.s001.docx]

**Supplementary Figure 1:** 1h uptake of ^3^H-maltose in bacteria (*E.coli*) and a mammalian cell line (EL 4)

**Supplementary Figure 2:** A) Bioluminescent image of a mouse bearing 10^6^ CFU of a bioluminescent strain of bacteria (see methods) on its right thigh B) A coronal slide from a micro PET/CT scan of the same mouse 1h after administration of 7.4MBq of 6-[^18^F]-fluoromaltose

**Supplementary Figure 3:** Time activity curve for mice (n=3) obtained from dynamic micro PET/CT showing distribution of 7.4MBq of 6-[^18^F]-fluoromaltose

in indicated organs

**Supplementary Figure 4:** Plot showing 30min uptake of 6-[^18^F]-fluoromaltose

in *E.coli* and residual activity observed at 30 minutes post efflux of tracer.
